# Difficult‐to‐treat patients with relapsed/refractory multiple myeloma: A review of clinical trial results

**DOI:** 10.1002/jha2.743

**Published:** 2023-08-02

**Authors:** Marc S. Raab, Elena Zamagni, Salomon Manier, Paula Rodriguez‐Otero, Fredrik Schjesvold, Annemiek Broijl

**Affiliations:** ^1^ Heidelberg Myeloma Center, Department of Medicine V University Hospital Heidelberg Germany; ^2^ Seragnoli Institute of Hematology Bologna University School of Medicine Bologna Italy; ^3^ Department of Hematology University Hospital Center of Lille Lille France; ^4^ Department of Hematology Clinica Universidad de Navarra Madrid Spain; ^5^ Oslo Myeloma Center, Department of Haematology Oslo University Hospital, Oslo, Norway, and KG Jebsen Center for B Cell Malignancies University of Oslo Oslo Norway; ^6^ Department of Hematology Erasmus MC Cancer Institute Rotterdam The Netherlands

**Keywords:** age, cytogenetics, extramedullary disease, frailty, renal impairment, RRMM

## Abstract

Overall outcomes for multiple myeloma have improved due to the availability of new therapies, but patients with relapsed/refractory multiple myeloma harbouring certain factors continue to pose a therapeutic challenge. These challenging features include high‐risk cytogenetics, renal impairment, patient characteristics such as age and frailty, and extramedullary disease. Prior refractory status and number of prior lines add further complexity to the treatment of these patients. While newer regimens are available and have suggested efficacy in these patient populations through subgroup analyses, differences in trial definitions and cut‐offs make meaningful comparisons difficult. This review aims to examine the available clinical trial data for patients with high‐risk cytogenetics, renal impairment, age and frailty and extramedullary disease.

## 1 INTRODUCTION

Multiple myeloma (MM) is a largely incurable disease that accounts for roughly 10% of haematologic malignancies. The main goal of treatment is to prolong the patient's long‐term outcomes [1]. Although significant improvements in overall survival have been made in the past 10–15 years due to immunomodulatory agents (IMiDs), proteasome inhibitors (PIs) and anti‐CD38 monoclonal antibodies (mAbs), this improvement has not been uniform and patients harbouring high‐risk features continue to do poorly [1, 2].

Patients with relapsed/refractory MM (RRMM) are a heterogenous group that represents a therapeutic challenge [1]. According to the International Myeloma Workshop Consensus Panel, they may be characterised as having primary refractory, refractory, relapsed, or both relapsed and refractory MM [1]. Refractoriness is often categorised by the number of drug classes involved, such as PIs, IMiDs, or anti‐CD38 mAbs [3]. When compared with patients with newly diagnosed MM (NDMM), those with RRMM respond less to IMiDs and PIs due to ongoing biological evolution of MM cells and their bone marrow environment that render the disease more resistant to treatment [1, 4]. As front‐line therapy often includes lenalidomide‐based regimens and treatment is planned until disease progression, many patients will progress under treatment becoming lenalidomide‐refractory [5]. Lenalidomide‐refractory patients are difficult to treat, have poorer survival outcomes, and experience worse outcomes with each successive line of therapy [6, 7].

Choice of treatment at the time of relapse is determined by patient and disease‐related factors and more importantly refractoriness and exposure to prior drugs [1]. At first relapse, the two most important considerations are whether the patient has lenalidomide‐refractory disease or not, and whether the disease is progressing on front‐line therapies that include anti‐CD38 monoclonal antibodies [3]. The International Myeloma Working Group (IMWG) has published treatment recommendations for these subgroups, based on the evidence from subgroup analyses of numerous clinical trials [3]. A number of high‐risk characteristics are considered for poor treatment outcome and shorter survival; among them are chromosomal abnormalities (CAs), renal impairment (RI), advanced age or frailty and presence of extramedullary disease (EMD). These subgroups are considered to be difficult to treat due to lower overall survival (OS) and/or response rates and a higher incidence of adverse events (AEs) than other groups, with a lack of evidence for recommended treatment regimens [2, 8–11]. This review will examine the current definitions and available clinical trial results, of patients with high‐risk cytogenetics, RI, older age and frailty, and EMD. Specific treatment recommendations for these challenging subgroups should be made by professional organizations and are beyond the scope of this review. Where possible, we refer to available guidelines or expert opinions.

## 2 CYTOGENETICS

### 2.1 Chromosomal changes are common in MM patients

High‐risk CAs in MM are detected by fluorescence in situ hybridisation (FISH), and are per IMWG guidelines — del(17p) with or without concomitant p53 mutation, translocation t(14;16) and t(4;14) [10, 12, 13, 14]. While t(4;14) has traditionally been associated with a poor outcome, recent data have indicated these patients are a heterogeneous group and prognosis may be driven by additional factors in the break‐point region [15]. Additional copies or gain of chromosome 1q (1q21+) is also a commonly observed CA in approximately 40% of patients with NDMM that increases to 70% in patients with relapsed MM [12, 14, 16, 17]. 1q21+ is classified as either gain1q21 (3 copies) or amp1q21 (≥4 copies), and typically worse prognosis for patients with amp1q21 than gain [18]. It also frequently coexists with other chromosomal abnormalities, which further worsens the prognosis for patients with 1q21+ [18]. Grading the impact of high‐risk markers in relation to respective therapies could be a new approach toward defining high‐risk cytogenetics in RRMM.

Along with other chromosomal changes such as t(14;20), presence of the aforementioned CAs are associated with lower survival in NDMM patients [10]. Some literature has indicated that t(14;16) may not be an independent prognostic factor, presenting often with at least one other high‐risk feature [19, 20]. Co‐segregation has also been reported for chromosome 1 abnormalities [21]. Patients with two or more high‐risk CAs on FISH are characterised as having ‘double‐hit’ MM, which portends poorer outcomes and high early mortality [22, 23].

### 2.2 Risk stratification of high‐risk cytogenetics definitions vary among studies and guidelines

It is known that patient OS varies according to cytogenetic status, among other factors [10, 24]. Risk classification based on cytogenetic profiling and patient subgroup stratification is thus important to evaluate new therapies and identify who is at higher risk for early disease progression and death [25, 26].

The IMWG currently recommends a minimum FISH panel to include t(4;14)(p16;q32), t(14;16)(q32;q23) and 17p13 deletions [24, 27]. In contrast, the Mayo Stratification for Myeloma and Risk‐adapted Therapy (mSMART) includes del(17p), t(4;14), t(14;16), t(14;20), and amp1q21 for FISH panel testing, while the European Society of Medical Oncology (ESMO) suggests testing for del17p, t(4;14), t(14;16), 1q21+ and t(11;14) [2, 28]. National Comprehensive Cancer Network (NCCN) considers t(4;14), t(14;16), del17p, 1q21+, MYC translocation, TP53 mutations, tetrasomies, and complex karyotypes as high‐risk cytogenetic abnormalities [29]. The Revised International Staging System (R‐ISS) for MM combines CA, serum albumin, β2 microglobulin, and lactate dehydrogenase (LDH) in its model for risk stratification. The high‐risk CA considered in the model are del(17p), translocation t(4;14) and t(14;16) [30]. The R‐ISS has recently been updated to the R2‐ISS, to allow for better stratification of intermediate‐risk patients [31]. The top predictors impacting OS and PFS were used to build an additive score, and included ISS, del(17p), LDH, t(4;14) and 1q+ [31]. The R2‐ISS was able to identify four well‐separated cohorts of patients and was validated in an independent cohort of patients [31].

A cytogenetic prognostic index scoring system was also developed by the Intergroupe Francophone du Myelome (IFM) that examined the prognostic impact of seven CAs — del(17p), t(4;14), del(1p32), gain1q21 and trisomies 3, 5 and 21 [32]. A higher prognostic index score was consistently associated with poor survival, and shorter survival was observed in patients classified in the intermediate‐ and high‐risk groups than those in the low‐risk group [32].

The poor prognostic impact of high‐risk cytogenetics is consistent across lines of therapy—in patients receiving first line therapy, median OS (mOS) is 48.9 months with high‐risk patients, and 77.0 months for standard‐risk patients. In second line, this decreases to 35.3 months and 54.8 months, respectively, and to 23.8 months and 46.3 months in third‐line [33].

Most RRMM clinical trials define high‐risk cytogenetics according to IMWG criteria, with ≥1 of del(17p), t(4;14) and/or t(14;16). 1q21+ is only considered as a high‐risk CA in the ICARIA‐MM, IKEMA and BOSTON trials [25, 34, 35, 36, 37, 38, 39, 40, 41, 42, 43].

The prognostic impact of cytogenetic abnormalities depends on the number of cells affected, and cut‐off values are not universally accepted [44]. Patients with del(17p) with a cut‐off value of >50% have been shown to display the worst prognosis, with a median PFS of 4.0 months compared to 24.0 months in those with del(17p) ≤50% [45]. Apart from the definition of high‐risk cytogenetics, these cut‐off values also vary among studies (Table 1) or may not be reported [25, 34, 38, 41, 42].

**TABLE 1:** Cut‐off values for affected cell ratios in Phase 3 MM trials.

Trial	Arms	del(17p)	t(4;14)	t(14;16)	amp1q21
**Pomalidomide‐based regimens**					
ICARIA‐MM [25]	Isa‐Pd versus Pd	≥50%	≥30%	≥30%	≥30%
APOLLO [35]	DPd versus Pd	NR	NR	NR	‐
ELOQUENT‐3 [39]	EPd versus Pd	NR	NR	NR	‐
**PI‐based regimens**					
IKEMA [34]	Isa‐Kd versus Kd	≥50%	≥30%	≥30%	≥30%
CANDOR [36]	DKd versus Kd	NR	NR	NR	‐
CASTOR [38]	DVd versus Vd	NR	NR	NR	‐
OPTIMISMM [40]	PVd versus Vd	NR	NR	NR	‐
BOSTON [41]	XVd versus Vd	≥10%	≥10%	≥10%	≥10%
**Lenalidomide‐based regimens**					
POLLUX [37]	DRd versus Rd	NR	NR	NR	‐
TOURMALINE‐MM1^a^ [42]	IRd versus Rd	≥5%	≥3%	≥3%	‐

Abbreviations: D, daratumumab; d, dexamethasone; E, elotuzumab; I, ixazomib; Isa, isatuximab; K, carfilzomib; NR, not reported; P, pomalidomide; R, lenalidomide; V bortezomib; X, selinexor.

^a^ Post‐hoc analyses applied different cut‐off values.

### 2.3 Impact of high‐risk cytogenetics in clinical trials

Subgroup analysis of phase 3 RRMM trials show that, in most cases, newer agents have provided clinical benefit to patients regardless of cytogenetic risk (Table 2; not intended for direct comparison). Of the listed trials, ICARIA‐MM, IKEMA, APOLLO, CANDOR, CASTOR, OPTIMISMM and ELOQUENT‐3 enrolled lenalidomide‐refractory or IMiD‐refractory patients [25, 34, 35, 36, 38, 39, 40]. Anti‐BCMA therapies are currently under development, and subgroup analyses of patients with high‐risk cytogenetics of phase 2 trials have been published, namely from the KarMMA trial (investigating idecabtagene vicleucel), DREAMM‐2 trial (belantamab mafodotin), and CARTITUDE‐1 (ciltacabtagene vicleucel) [43, 46, 47, 48, 49]. The results from the analyses from these 3 trials also reflect a clinical benefit to patients regardless of cytogenetic risk, although again, patients with standard risk have better PFS [43, 46, 47, 48, 49].

**TABLE 2:** PFS in Phase 3 MM trials in standard‐risk and high‐risk patients.

		ITT population		Cytogenetics group PFS (months)		
Trial	Arm	PFS (months)	Hazard ratio (95% CI)	HR	SR	Hazard ratio (95% CI)
**Pomalidomide‐based regimens**						
ICARIA^a^ [25, 97]	Isa‐Pd	11.1	0.60 (0.46‐0.78)	7.5	11.6	HR: 0.66 (0.33–1.28)
	Pd	5.9		3.7	7.4	SR: 0.62 (0.42–0.93)
APOLLO^a^ [35]	DPd	12.4	0.63 (0.47‐0.85)	5.8	21.0	HR: 0.85 (0.49–1.44)
	Pd	6.9		4.0	7.4	SR: 0.51 (0.32–0.81)
**PI‐based regimens**						
IKEMA^a^ [98, 99]	Isa‐Kd	35.7	0.58 (95.4% CI 0.42‐0.79)	NR	18.2	HR: 0.72 (0.36–1.45)
	Kd	19.2		NR	19.5	SR: 0.44 (0.27–0.73)
CASTOR^b^ [38]	DVd	16.7	0.31 (0.25‐0.40)	12.6	16.6	HR: 0.41 (0.21–0.83)
	Vd	7.1		6.2	6.6	SR: 0.26 (0.19–0.37)
OPTIMISMM^c^ [40, 100]	PVd	11.2	0.61 (0.49‐0.77)	14.7	–	HR: 0.39 (0.13–1.17)
	Vd	7.1		9.9	–	
BOSTON^c^ [41, 101]	XVd	13.9	0.70 (0.53‐0.93)	12.9	16.6	HR: 0.67 (0.45–0.98)
	Vd	9.5		8.1	9.7	SR: 0.63 (0.42–0.95)
CANDOR^d^ [102, 103]	DKd	28.6	0.59 (0.45‐0.78)	11.2	NR	HR: 0.56 (0.34–0.93)
	Kd	15.2		7.4	16.6	SR: 0.56 (0.39–0.80)
**Lenalidomide‐based regimens**						
POLLUX^b^ [37, 104]	DRd	NR	0.37 (0.27‐0.52)	26.8	NR	HR: 0.34 (0.16–0.72)
	Rd	18.4		8.3	18.6	SR: 0.43 (0.32–0.57)
TOURMALINE‐MM1^a^, ^c^ [42, 105]	IRd	20.6	0.74 (0.59‐0.94)	21.4	20.6	HR: 0.54 (0.32–0.92)
	Rd	14.7		9.7	15.6	SR: 0.64 (0.46–0.89)

Abbreviations: d, dexamethasone; D, daratumumab; HR, high‐risk; I, ixazomib; Isa, isatuximab; K, carfilzomib; NA, not applicable; NR, not reached; P, pomalidomide; PFS, progression‐free survival; R, lenalidomide; SR, standard‐risk; V, bortezomib; X, selinexor.

^a^ Prespecified subgroup analysis.

^b^ Exploratory subgroup analysis.

^c^ Post‐hoc subgroup analysis.

^d^ Analysis type not specified.

–Not reported.

However, PFS for patients with high‐risk cytogenetics remains shorter than for patients with standard‐risk MM, indicating that better treatment options are still needed for this important subgroup. It is also important to note that the subgroup analysis shown in Table 2 is limited by sample size.

Achievement of minimal residual disease (MRD) negativity has also emerged as a strong indicator of prognosis in patients, although most of the literature is focused on NDMM patients [50]. Patients are often assessed for MRD status when they achieve complete response (CR), but also in very good partial response (VGPR).[51] MRD is typically evaluated by next‐generation flow or next‐generation sequencing (NGS) at a minimum sensitivity level of 10^−5^ [52, 53, 54]. Regimens providing high MRD negativity rates are thus of value in this setting.

Few trials have published MRD negativity data for subgroups of patients with high‐risk CA. A subgroup analysis of POLLUX, investigating DRd versus Rd in RRMM patients found MRD negativity at a sensitivity of 10^−5^ (by NGS) in high‐risk patients was only achieved with DRd, but only one of nine patients was able to sustain the response [37]. In a subgroup analysis of CASTOR, rates of MRD negativity at the 10^−5^ sensitivity threshold (NGS) in patients achieving CR were higher with DVd compared with Vd in both patients with standard‐risk cytogenetics (11% vs. 3%; *p* = 0.0091) and high‐risk cytogenetics (15% vs. 0%; p = 0.0271) [38]. MRD negativity was sustained for at least 12 months in two patients (1%) with standard‐risk cytogenetics and three (8%) patients with high‐risk cytogenetics in the DVd group, compared with none in both cytogenetic risk categories in the Vd group [38].

Subgroup analysis of safety in RRMM clinical trials indicates that patients with high‐risk status tend to have a higher incidence of Grade ≥3 treatment‐emergent adverse events (TEAEs) compared with their standard‐risk counterparts. However, this observation may be driven by differences in sample size across trials, and due to few trials reporting safety analyses in this subgroup. Subgroup safety analysis was not performed in APOLLO, CANDOR or ELOQUENT‐3.

### 2.4 Summary

There is a need to harmonise the definition of high‐risk cytogenetics and the cut‐off values used in future clinical trials. Heterogeneity in the definition between trials adds to the difficulty of cross‐trial comparisons and ability to make treatment recommendations. Patients’ cytogenetic status should be determined at both diagnosis and relapse. More evidence and consistent definitions are needed in order for risk status to guide treatment decisions in the future. Patients with high‐risk cytogenetics can benefit from the addition of a mAb to standard‐of‐care backbones. The current best treatment options for these patients, and even those with standard‐risk, should be a triplet therapy, either combining PI and IMiD with dexamethasone, or a mAb with PI or IMiD with dexamethasone. Quadruplet regimens and emerging treatments, including CAR‐T cell therapies and bispecific antibodies, may also provide a benefit for patients with high‐risk cytogenetics. However, treatment regimens that consistently overcome the poor prognosis of high‐risk RRMM remain to be found.

## 3 RENAL IMPAIRMENT

### 3.1 RI is common in MM and is associated with poor survival

RI and worsening renal function is a known high‐risk factor for both NDMM and RRMM that is associated with poor OS [55, 56]. Between 20% and 50% of patients have RI at diagnosis, which may be caused by cast nephropathy, light chain deposition disease, AL amyloidosis, and hypercalcaemia [55, 57].

Novel agents have significantly improved the OS of patients with RI from 21 months to 60 months [58]. Bortezomib‐based regimens are the treatment of choice for MM patients with RI, having demonstrated reversal of renal dysfunction and significant rates of renal responses [59].

### 3.2 The need for standardisation of RI in clinical trials and the impact of novel treatments on patients with RI

RI is not reported in a standard format—definitions differ between trials. While RI is reported as estimated glomerular filtration rate (eGFR; mL/min/1.73 m^2^), different formulas are available for calculating eGFR [55]. Cut‐offs may also vary according to the trial, as seen in Table 3.

**TABLE 3:** PFS hazard ratio based on baseline RI status in Phase 3 MM trials.

Trial	Arms	Minimum eGFR per inclusion criteria (mL/min)	Baseline eGFR (mL/min)	PFS HR (95% CI), or *p* value
**Pomalidomide‐based regimens**				
**ICARIA** ^a^ [**63**, **106**]	Isa‐Pd versus Pd	≥30	<60	0.50 (0.30; 0.85)
			>60	0.58 (0.38; 0.88)
**PI‐based regimens**				
**IKEMA** **^a^** [**64**]	Isa‐Kd versus Kd	≥15	≤60	0.27 (0.11; 0.66)
			>60	0.63 (0.39; 1.00)
**BOSTON** **^b^**[**107**]	XVd versus Vd	≥20	<40	0.62; p = 0.129
			40‐60	0.49; p = 0.028
			>60	0.71; p = 0.019
			**Baseline CrCl (mL/min)**	
**PI‐based regimens**				
**CANDOR** **^a^** [**36**]	DKd versus Kd	≥20	≥15 to <50	0.44 (0.19; 1.00)
			≥50 to <80	0.65 (0.36; 1.15)
			≥80	0.68 (0.44; 1.03)
**CASTOR** **^a^**[**108**]	DVd versus Vd	>20	≤60	0.55 (0.30; 1.02)
			>60	0.30 (0.20; 0.44)
**OPTIMISMM** **^c^** [**66**]	PVd versus Vd	≥30	<60	0.67 (0.34; 1.34)
			≥60	0.45 (0.27; 0.76)
**ENDEAVOR** **^d^** [**62**]	Kd versus Vd	≥15	≥15 to <50	0.49 (0.32; 0.76)
			≥50 to <80	0.48 (0.35; 0.65)
			≥80	0.60 (0.43; 0.83)
**Lenalidomide‐based regimens**				
**POLLUX** **^c^** [**109**]	DRd versus Rd	>30	≤60	0.41 (0.26; 0.65)
			>60	0.44 (0.33; 0.57)
**ELOQUENT‐2** **^c^** [**110**]	ERd versus Rd	≥30	<60	0.56 (0.39; 0.82)
			≥60	0.74 (0.58; 0.94)

Abbreviations: CI, confidence interval; CrCl, creatinine clearance; D, daratumumab; d, dexamethasone; E, elotuzumab; eGFR, estimated glomerular filtration rate; HR, hazard ratio; Isa, isatuximab; K, carfilzomib; P, pomalidomide; PFS, progression‐free survival; R, lenalidomide; V, bortezomib; X, selinexor.

^a^ Prespecified subgroup analysis.

^b^ Post‐hoc analysis.

^c^ Analysis type not specified.

^d^ Post‐hoc exploratory subgroup analysis.

The available subgroup analyses of phase 3 RRMM trials show that regardless of CrCl or eGFR (min/mL) at baseline, PFS is improved with newer agents (Table 3; not intended for comparison). However, many trials may exclude patients with moderate renal failure or worse and thus may not be an accurate reflection of the efficacy of newer agents in the RI subpopulation and pose a challenge in real‐world use.

### 3.3 Renal response assessment in trials

Renal response can be used to measure when RI is improved by treatment [55]. Complete renal response (CrR) is defined by IMWG as an increase in baseline eGFR to ≥60 mL/min, while partial renal response is defined as an increase of eGFR from a baseline of <15 mL/min to 30–59 mL/min, and minor renal response is defined as an increase from <15 mL/min to 15–20 mL/min, or if baseline eGFR is 15–29 mL/min, an increase to 30–59 mL/min [57]. With the latter definition of minor renal response, patients are unable to achieve partial response by definition, illustrating the need for revised renal response criteria. Patients with severe RI who achieve CrR have improved survival (27 months) over those who do not achieve renal response (18 months) [57]. However, as patients receive additional lines of therapy, survival may worsen [60]. Early and effective intervention is thus necessary to prevent deterioration of renal function in many patients [61].

Few trials have reported renal response data, and renal responses are also not standardised to IMWG criteria. In the ENDEAVOR study, CrR was defined as CrCl ≥60 mL/min if patients had these levels in at least two consecutive visits, if the patient's baseline CrCl value was <50 mL/min [62]. In ICARIA‐MM, renal response was defined as eGFR improvement from <50 mL/min/1.73 m^2^ at baseline to ≥60 mL/min/1.73 m^2^ in at least one assessment [63]. Response was considered durable if it lasted ≥60 days. Renal response criteria in IKEMA were similarly assessed [64].

In ICARIA‐MM, 71.9% of patients in the Isa‐Pd arm achieved CrR versus 38.1% in those receiving Pd. Durable CrR (response that lasted ≥60 days) was observed in 31.3% and 19.0% of these patients, respectively [63]. Patients receiving Isa‐Pd had a time to renal response of 3.4 months versus 7.3 months in those receiving Pd [63]. In the IKEMA trial, CrR was achieved in 52.0% and 30.8% of Isa‐Kd and Kd patients, respectively, while durable CrR was observed in 32.0% and 7.7% of corresponding patients [64]. Time to complete renal response was 7.8 months with Isa‐Kd, and NR with Kd alone [65]. The ENDEAVOR trial reported 15.3% and 14.1% CrR rates with Kd and Vd, respectively, and corresponding time to CrR was 1.9 months and 1.5 months [62]. In OPTIMISMM, only median time to first improvement in renal function was reported, which was 3.1 months with PVd and 3.6 months with Vd (*p* = 0.859) [66].

### 3.4 Impact of RI on treatment options

RI also impacts the treatments available to the patient; for instance, lenalidomide is excreted through the kidneys and requires dose adjustments according to degree of RI, and NDMM patients with RI may not receive high‐dose treatment with autologous stem cell transplant (ASCT) due to the risk of toxicity [55]. IMWG has defined dose adjustments for patients with CrCl 30–59 mL/min, CrCl 15–29 mL/min, CrCl <15 mL/min, or on dialysis for treatment with melphalan and lenalidomide [55]. The IMWG recommended dose adjustments of MM therapies due to RI are seen in Table 4. Monoclonal antibodies such as daratumumab, isatuximab and elotuzumab require no dose modifications in patients with CrCl 30–59 mL/min, CrCl 15–29 mL/min, CrCl <15 mL/min, or on dialysis for treatment [67, 68, 69]. In patients with CrCl >60 mL/min, daratumumab should be administered at a dosage of 16 mg/kg (IV) or 1800 mg (subcutaneous), and isatuximab and elotuzumab at a dosage of 10 mg/kg [67, 68, 69]. Ixazomib should be taken at a dose of 4 mg once weekly (QW) in those with CrCl >60 mL/min and 30–50 mL/min, and 3 mg QW in patients with CrCl 15–29 mL/min, <15 mL/min, and on dialysis without regard to timing of dialysis [70].

**TABLE 4:** Dose modifications for drugs used for the management of patients with MM with RI[55].

Drug	Cr Cl >60 mL/min	CrCl 30–59 mL/min	CrCl 15–29 mL/min	CrCl <15 mL/min	On dialysis
Dexamethasone	20–40 mg	No dose modification needed	No dose modification needed	No dose modification needed	No dose modification needed
Melphalan	Oral melphalan 0.15 to 0.25 mg/kg/d for 4–7 days	Oral melphalan reduced 25% (0.11–0.19 mg/kg/d for 4–7 days)	Oral melphalan reduced 25% (0.11–0.19 mg/kg/d for 4–7 days)	Oral melphalan reduced 50% (0.0175–0.125 mg/kg/d for 4–7 days)	Oral melphalan reduced 50% (0.0175‐0.125 mg/kg/d for 4–7 days)
	High‐dose melphalan 200 mg/m^2^	High‐dose melphalan 140 mg/m^2^	High‐dose melphalan 140 mg/m^2^	High‐dose melphalan 140 mg/m^2^	High‐dose melphalan 140 mg/m^2^
Bortezomib	1.3 mg/m^2^ on days 1, 4, 8, and 11, or weekly regimens	No dose modification needed	No dose modification needed	No dose modification needed	No dose modification needed
Thalidomide	50–200 mg/d	No dose modification needed	No dose modification needed	No dose modification needed	No dose modification needed
Lenalidomide	25 mg/d	10 mg per d, can be increased to 15 mg/d if no toxicity occurs	15 mg once every other d, can be adjusted to 10 mg/d	5 mg/d	5 mg/d
Carfilzomib	20 mg/m^2^ cycle 1; 27 mg/m^2^ cycle 2, and on	No dose modification needed	No dose modification needed	No dose modification needed	No dose modification needed
Doxorubicin	According to regimen	No dose modification needed	No dose modification needed	No dose modification needed	No dose modification needed
Cyclophosphamide	According to regimen	No dose modification needed	No dose modification needed	No dose modification needed	No dose modification needed
Pomalidomide	4 mg/d	No dose modification needed for CrCl ≥ 45 mL/min	Ongoing studies will clarify if modification is needed	Ongoing studies will clarify if modification is needed	Ongoing studies will clarify if modification is needed

*Note*: Dimopoulos MA, Sonneveld P, Leung N, Merlini G, Ludwig H, Kastritis E, et al. International Myeloma Working Group recommendations for the diagnosis and management of myeloma‐related renal impairment. J Clin Oncol. 2016;34(13):1544‐57. https://doi.org/10.1200/JCO.2015.65.0044. Copyright © 2022 American Society of Clinical Oncology. All rights reserved.

Abbreviations: CrCl, creatinine clearance; d, day; IV, intravenous.

As lenalidomide is renally cleared, a phase 1/2 trial has explored the maximum tolerated dose and efficacy of lenalidomide in patients with varying degrees of renal impairment [71]. This trial recommended full dose lenalidomide 25 mg daily for 21 days out of 28 (21/28) for patients with CrCl ≥30 mL/min. In patients with CrCl <30 mL/min, regardless of whether they were on dialysis, lenalidomide can be given at a dose of at least 15 mg daily 21/28, and can be given on a daily dosing regimen, in contrast to three times weekly [71].

### 3.5 Summary

It is clear that patients with RI have historically poor outcomes. RI should not preclude effective and timely treatment, as efficient front‐line treatment is key to preventing deterioration of renal function and improving survival, particularly as rapid and sustained renal response is associated with improved survival [57]. The available literature has demonstrated that newer regimens are efficacious in patients with RI, with improved PFS compared to historical outcomes, and with good tolerability that may not require dose modifications [55]. Renal response should also be standardised to IMWG criteria, whilst ensuring it is also reported in clinical trial data. Few trials have reported their renal response data, but isatuximab in the ICARIA‐MM and IKEMA trials have shown newer regimens can lead to improved CrR and durable CrR rates, with quick times to renal response [63, 64]. Where possible, renal response should be incorporated into the clinical trial design and subgroup analyses published in order to elucidate the best treatment for patients with RI.

## 4 PATIENT PERFORMANCE (AGE AND FRAILTY)

### 4.1 MM is a disease of the elderly, and poor survival correlates with age

MM is primarily a disease of the elderly and is most frequently diagnosed in people aged 65–74 years, with a median age of diagnosis of 69 years [72]. Data from the SEER database in the United States have shown overall, two of three of patients are diagnosed above the age of 65 —31.5% of new cases are aged 65–74, 23.5% aged 75–84 years and 9% aged >84 [72]. Older age is associated with functional decline, comorbidities and organ dysfunction [73].

Age is correlated with poor survival; in patients diagnosed after 2010, 5‐year survival was 65% for patients younger than 65 (64.7%; 95% CI: 63.4–65.9), 48% (48.1%; 95% CI: 46.7–49.6) for patients between 65 and 74 years old, and 31% (30.6%; 95% CI: 29.3–32.0) for those older than 75 years [74]. The risk of dying in the first year was higher in patients aged 75–90 years than that for patients aged 65 years or younger [74]. Newer therapies have thus changed the prognosis of this population, although non‐frail patients still have better prognosis [75]. The median number of treatment lines decreases with increasing age, which may also explain the shorter overall survival in the elderly [76].

### 4.2 Treatment efficacy in clinical trials according to age group

**FIGURE 1:**
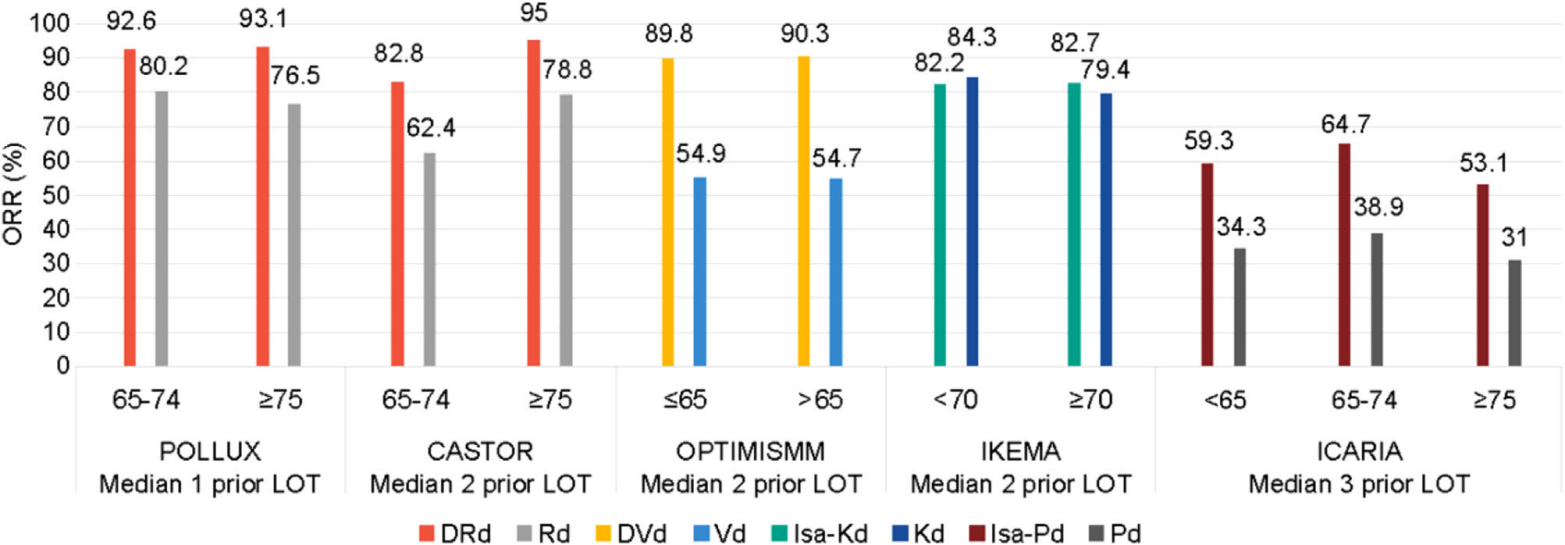
Overall response rate based on age in multiple myeloma trials. Abbreviations: D, daratumumab; d, dexamethasone; Isa, isatuximab; K, carfilzomib; LOT, line of therapy; ORR, overall response rate; P, pomalidomide; R, lenalidomide; V, bortezomib.

Phase 3 clinical trials of newer regimens, including anti‐CD38 mAbs, have demonstrated prolonged PFS and demonstrated efficacy across various age subgroups in elderly people, as seen from Table 5 and Figure 1. In these subgroup analyses, lenalidomide‐refractory patients were reported in OPTIMISMM and ICARIA‐MM and consisted of 51.0%–60.9% and 1.5%–21.9% of the populations, respectively.

**TABLE 5:** PFS hazard ratio based on age in Phase 3 MM trials.

Trial	Arms	Age group (years)	PFS (months)	PFS HR (95% CI), or *p* value
**Pomalidomide‐based regimens**				
ICARIA‐MM^a^ [81]	Isa‐Pd versus Pd	<65	11.5 versus 5.0	0.66 (0.40–1.07)
		65–74	11.6 versus 8.6	0.64 (0.39–1.06)
		≥75	11.4 versus 4.5	0.48 (0.24–0.95)
APOLLO^a^ [35]	DPd versus Pd	<65	9.2 versus 5.8	0.69 (0.44–1.09)
		≥65	14.2 versus 7.0	0.55 (0.38–0.81)
**PI‐based regimens**				
IKEMA^b^ [34]	Isa‐Kd versus Kd	<65	NR versus NR	0.64 (0.37–1.11)
		≥65	NR versus 17.2	0.43 (0.25–0.74)
CANDOR^a^ [36]	DKd versus Kd	≤65	–	0.57 (0.38–0.86)
		>65	–	0.76 (0.48–1.22)
CASTOR^c^ [80]	DVd versus Vd	65‐74	18.9 versus 6.1	0.25 (0.16–0.40); *p* < 0.0001
		≥75	17.9 versus 8.1	0.26 (0.10–0.65); *p* = 0.0022
OPTIMISMM^c^, ^d^ [40]	PVd versus Vd	≤65	22.0 versus 13.1	0.49 (0.26–0.93); *p* = 0.0258
		>65	17.6 versus 9.9	0.57 (0.34–0.97); *p* = 0.0369
ASPIRE^b^ [111]	KRd versus Rd	<70	28.6 versus 17.6	0.70 (0.56–0.88)
		≥70	23.8 versus 16.0	0.75 (0.53–1.08)
ENDEAVOR^b^ [112]	Kd versus Vd	<65	NR versus 9.5	0.58 (0.44–0.77)
		65‐74	15.6 versus 9.5	0.53 (0.38–0.73)
		≥75	18.7 versus 8.9	0.38 (0.23–0.65)
ARROW^c^ [113]	Kd 70 mg/m^2^ QW versus Kd 27 mg/m^2^ BIW	<65	12.2 versus 5.6	0.60 (0.42–0.86)
		65‐74	9.2 versus 8.4	0.84 (0.58–1.23)
		≥75	12.2 versus 9.5	0.80 (0.43–1.48)
**Lenalidomide‐based regimens**				
POLLUX^c^[80]	DRd versus Rd	65‐74	NR versus 17.1	0.40 (0.27–0.60); *p* < 0.0001
		≥75	28.9 versus 11.4	0.27 (0.10–0.69); *p* = 0.0042

Abbreviations: BIW, twice weekly; CI, confidence interval; D, daratumumab; d, dexamethasone; HR, hazard ratio; Isa, isatuximab; K, carfilzomib; NR, not reached; P, pomalidomide; PFS, progression‐free survival; QW, once weekly; R, lenalidomide; V, bortezomib.

^a^ Prespecified subgroup analysis.

^b^ Post‐hoc subgroup analysis.

^c^ Analysis type not specified.

^d^ PFS in patients with 1 prior line of therapy.

Few trials have analysed treatment tolerability by age. A summary of safety data available from phase 3 clinical trials by age can be seen in Table 6. New agents have shown a tolerable safety profile, although the incidence of Grade 3/4 TEAEs is increased in older (≥75 years) patients. Only ICARIA‐MM and IKEMA reported the incidence of TEAEs leading to discontinuation by age group, which was low, ranging from 3.0 to 15.6% in the Isa‐Pd arm, and 7.1%–11.8% in the Isa‐Kd arm, respectively. The phase 2 KarMMA trial of idecabtagene vicleucel, has also published a safety subgroup analysis by age, with cutoffs of ≥65, and ≥70 [77]. The incidence of grade ≥3 cytokine release syndrome was higher in those aged ≥70 (10.0% vs. 4.0%) while the incidence of grade ≥3 neurotoxicity was higher in patients aged ≥65 (9.0% vs. 5.0%) [77].

**TABLE 6:** Safety subgroup analysis by age.

Trial	Arms	Age (years)	Grade 3/4 TEAEs (%)	Grade 3/4 neutropenia (%)
**Pomalidomide‐based regimens**				
ICARIA‐MM [81]	Isa‐Pd versus Pd	<65	85.2 versus 64.7	44.4 versus 26.5
		65‐74	84.8 versus 75.5	45.5 versus 32.1
		≥75	93.8 versus 75.0	50.0 versus 46.4
**PI‐based regimens**				
IKEMA [83]	Isa‐Kd versus Kd	<70	71.4 versus 63.6	NR
		≥70	90.2 versus 76.5	NR
CASTOR [80]	DVd versus Vd	65‐74	81.9 versus 69.8	16.0 versus 3.5
		≥75	90.0 versus 74.3	0 versus 2.9
OPTIMISMM [82, 84]	PVd versus Vd	≤65	NR	49.0 versus 6.3
		>65	NR	25.8 versus 12.9
**Lenalidomide‐based regimens**				
POLLUX [80]	DRd versus Rd	65‐74	91.9 versus 82.4	55.3 versus 39.8
		≥75	86.2 versus 77.1	44.8 versus 31.4

Abbreviations: D, daratumumab; d, dexamethasone; Isa, isatuximab; K, carfilzomib; NR, not reported; P, pomalidomide; R, lenalidomide; TEAE, treatment‐emergent adverse event; V, bortezomib;

### 4.3 The impact of frailty on treatment outcomes

Frailty must also be considered in MM patients and is a predictor of treatment outcomes and treatment toxicity [8]. The IMWG has developed a frailty score that considers age, functional status and comorbidities to predict patient survival and treatment toxicity [8, 73]. It was developed to be used in clinical trials to define patient frailty as it led to drug dose reduction and treatment discontinuation [8]. However, reduction and discontinuation also leads to worse outcomes [8]. In frail NDMM patients, trials have investigated dose‐adjusted lenalidomide‐dexamethasone combinations where dexamethasone is interrupted, and novel combinations such as daratumumab‐ixazomib‐low dose dexamethasone [78, 79]. Available data from phase 3 clinical trials investigating novel regimens in RRMM with frailty subgroup analysis can be seen in Table 7.

**TABLE 7:** PFS hazard ratio based on frailty status in phase 3 MM trials.

Trial	Arms	Frailty status	PFS HR (95% CI), or *p* value
**Pomalidomide‐based regimens**			
ICARIA‐MM^a^ [114]	Isa‐Pd versus Pd	Yes	0.81 (0.45–1.48)
		No	0.49 (0.33–0.73)
**PI‐based regimens**			
ASPIRE^a^ [115]	KRd versus Rd	Yes	0.78 (0.54–1.12)
		No	0.70 (0.49–1.01)
ENDEAVOR^a^ [115]	Kd versus Vd	Yes	0.50 (0.36–0.68)
		No	0.51 (0.33–0.79)
ARROW^a^ [115]	Kd 70 mg/m^2^ QW versus Kd 27 mg/m^2^ BIW	Yes	0.76 (0.49–1.16)
		No	0.53 (0.33–0.86)

^a^ Post‐hoc subgroup analysis

Abbreviations: BIW, twice weekly; CI, confidence interval; D, daratumumab; d, dexamethasone; HR, hazard ratio; Isa, isatuximab; K, carfilzomib; P, pomalidomide; PFS, progression‐free survival; QW, once weekly; R, lenalidomide; V, bortezomib.

The results displayed in Table 7 show that newer regimens are effective in both frail and fit RRMM patients. However, this may not be an accurate reflection of frail patients, as truly frail patients are likely excluded from clinical trials due to inclusion criteria. Frailty may thus be more important than age in determining treatment outcomes. Increasing frailty score is also associated with a greater incidence of treatment discontinuation and grade 3–4 non‐haematologic AEs [8].

### 4.4 Summary

The literature indicates elderly and frail patients have adverse survival outcomes and are more vulnerable to treatment‐related toxicities compared with young, fit patients. Frailty may be a stronger determinant of outcomes than chronological age. Newer regimens, including anti‐CD38 mAbs are feasible and well tolerated in both elderly and frail patients, with infection rates broadly similar to young, fit patients. However, the publishing of subgroup safety analyses of clinical trials in elderly populations should be encouraged, given the few currently available [77, 80, 81, 82, 83, 84]. Frail patients should still receive the best appropriate treatment with dose reductions to be evaluated over time in each patient to avoid under‐treatment. If a triplet regimen is considered for frail patients, monoclonal antibodies appear to be a preferred combination partner due to the good tolerability in this patient population. However, it should be noted that phase 3 studies may include an unrepresentative patient population, and up to 40% of patients with MM in the real world do not meet criteria for inclusion in phase 3 studies [85]. Patients may be ineligible due to various reasons, including poor performance status or inadequate organ function, and are thus underrepresented in clinical trials [85]. Clinical trials thus have a lack of representative data about the efficacy of treatments in real‐world patients and more evidence should be generated to reflect the actual population.

## 5 EXTRAMEDULLARY DISEASE

### 5.1 Paraskeletal plasmacytomas versus extramedullary disease

While plasma cell proliferation is usually restricted to the bone marrow in MM, clonal plasma cells may escape the bone marrow, leading to EMD [11]. The reported incidence of EMD involvement at diagnosis ranges from 1.7% to 4.5% [86]. At relapse, the incidence of EMD increases from 3.4% up to 10% [86]. EMD is also often associated with high‐risk cytogenetics [86].

EMD may be of two types – paraskeletal plasmacytomas, that is, growth of an intramedullary lesion continues by breaking through the cortical bone, and extramedullary plasmacytomas, that manifest and grow independently of any bone or bone marrow structures [11, 86]. Paraskeletal plasmacytomas have a better prognosis as cells are more differentiated than EMD [86]. Extramedullary plasmacytomas are an aggressive form of MM that is usually associated with high‐risk chromosomal changes, increased proliferation, evasion of apoptosis, and resistance to therapies, and the worst prognosis stems from central nervous system (CNS) involvement [11]. The survival rates for patients with extramedullary relapse are significantly lower than in patients with paraskeletal relapse (30 vs. 45 months; *p* = 0.022), and soft tissue involvement at any point is associated with poorer survival [86, 87].

### 5.2 Patients with EMD have poor survival

Patients with EMD had a significantly worse 3‐year PFS of 39.9% (95% CI: 30.3–49.5) in comparison to patients without EMD (47.9%, *p* = 0.001) and patients with paraskeletal involvement (50.0%, *p* = 0.007), and a significantly worse 3‐year OS of 58.0% versus 80.1% and 77.7%, respectively (95% CI: 48.1–67.9; *p* < 0.001) [88]. When comparing the MM group without EMD to those with EMD, a similar 3‐year PFS of 49.4% (95% CI: 44.6–54.3; *p* = 0.36) was observed with one involved site, while multiple involved sites showed a worse PFS of 22.7% (95% CI: 5.2–40.2; *p* = 0.001) [88]. Patients with one and multiple involved sites of EMD showed worse 3‐year OS rates of 73.5% (95% CI: 69.2–77.7; *p* < 0.001) and 71.4% (95% CI: 55.1–87.7; *p* = 0.05) in comparison to patients without EMD (80.1%) [88]. In patients with RRMM, EMD is associated with significantly shorter OS than those without [11]. If CNS involvement is present, median overall survival can range from 2 to 3 months, and novel agents do not seem to improve survival [89, 90]. In a study of carfilzomib‐containing therapies given to 45 patients with RRMM and EMD, EMD without adjacency to bone was associated with a significantly shorter PFS (*p* = 0.004) and OS (*p* = 0.04) compared with paraosseous lesions [11].

### 5.3 EMD detection is not standard

As with RI and cytogenetics, there is no standard detection method for EMD, and several different methods to detect extramedullary involvement in MM have been used in the published literature [11]. Magnetic resonance imaging (MRI) is the gold standard for detecting bone marrow involvement in MM and to study the CNS[91]. MRI is preferred for detecting paraskeletal plasmacytomas, while FDG‐PET/CT is preferred for extramedullary disease. FDG‐PET/CT should be performed in patients with clinical symptoms, patients considered at high risk, and at time of biochemical relapse in patients with a history of extramedullary plasmacytomas [86]. Moreover, functional imaging techniques are strongly recommended for the evaluation of response after treatment (when possible the same as up‐front), in order to clearly distinguish active from old lesions. A revision of the definition of plasmacytoma response after therapy is currently under discussion.

### 5.4 Response rates in clinical trials in patients with EMD

Available subgroup analyses of patients with EMD in randomized clinical trials are sparse, with many only reporting efficacy as response rates. Only two phase 3 trials, ICARIA‐MM and IKEMA, have investigated EMD in a subgroup analysis. In ICARIA‐MM, the ORR was 50% with Isa‐Pd and 10% with Pd alone, and VGPR occurred in 21.4% and 10% of Isa‐Pd and Pd patients, respectively. 2 of 14 patients who presented with plasmacytomas at baseline with VGPR in the Isa‐Pd arm showed complete remission at cycle 3 and significant reduction at cycle 4 of the extramedullary lesions, respectively, versus 0 in the Pd arm [92]. The IKEMA study also evaluated the safety of Isa‐Kd versus Kd in patients with relapsed MM and pre‐existing soft tissue plasmacytomas, and observed improved VGPR or better with Isa‐Kd versus Kd (33.3% vs. 14.3%) [93].

ORR has been reported in several phase 2 trials that have performed subgroup analyses on enrolled patients with EMD. In the DREAMM‐2 study, 9.1% (95% CI: 1.1–29.2) of MM patients with EMD who were treated with 2.5 mg/kg belantamab mafodotin had an ORR of 9.1% and 5.6% with the 2.5 mg/kg and 3.4 mg/kg doses in patients with EMD was reported, compared to 37.3% and 40.7% in those without EMD [94]. In the phase 2 STORM study, 5 patients had objective responses, based upon para‐protein and plasmacytoma reductions according to IMWG criteria — 1 VGPR and 4 partial responses (PR), for an ORR of 18.5% [95]. With ide‐cel monotherapy in the KarMMA trial, ORR was 70% in patients with EMD, and 76% in patients without, while CR rates were 24% and 39%, respectively [43]. In CARTITUDE‐1, the overall ORR was 97.9% (95% CI: 92.7–99.7), compared to 100.0% (95% CI: 82.4–100) in those with plasmactyomas [48]. In MajesTEC‐1, teclistamab monotherapy in patients without EMD plasmacytomas had a higher ORR than those with 1 or more plasmacytomas [96].

### 5.5 Summary

In conclusion, the presence of EMD is associated with reduced PFS and OS, with soft tissue plasmacytomas conveying a poorer prognosis than bone‐related plasmacytomas. FDG‐PET/CT is the recommended whole‐body technique for suspected soft tissue involvement; alternatively, MRI can be used, in particular, to evaluate lesions in the axial skeleton/CNS. IMWG Uniform Response Criteria require a ≥50% decrease in soft tissue plasmacytomas for PR and the disappearance of soft tissue plasmacytomas for CR. Updated definitions of plasmacytoma response, involving functional imaging, are currently under development and should be included in future clinical trials. While there are currently limited data for the efficacy of newer regimens in EMD, the available subgroup analyses suggest that newer regimens such as the anti‐CD38 mAb isatuximab may provide a benefit in patients with EMD through improved responses. More evidence from randomized clinical trials with larger sample sizes is needed in order to make treatment recommendations for RRMM patients with EMD.

## 6 CONCLUSION

Challenging factors such as high‐risk cytogenetics, RI, age and frailty, and EMD remain unmet needs in the treatment of RRMM. A list of most recent recommendations from international organisations and experts for these subgroups can be found in Table 8. This review has found that although some subgroup analyses of clinical trials investigating novel regimens or treatments have indicated benefits to these challenging patients, more data with larger sample sizes or meta‐analyses are needed to confirm these findings, particularly in the case of EMD, with few patients included in clinical trials. The tables summarize the currently available clinical phase 3 trial data for these subgroups and highlight where more evidence is needed. Further, few trials in these populations include lenalidomide‐refractory patients and given most patients will be exposed to lenalidomide early in their treatment course, a high proportion of RRMM patients are lenalidomide‐refractory. Prior treatment lines and lenalidomide‐refractoriness should also be considered when determining treatment for these difficult‐to‐treat subgroups. Anti‐CD38 and emerging treatments, including CAR‐T cell therapies and bispecific antibodies towards novel targets, may provide a benefit for patients with high‐risk cytogenetics or EMD. When designing clinical trials, standardised definitions and cut‐off values for patient characteristics such as high‐risk cytogenetics or RI should also be used whenever possible in order to improve data interpretation and define treatment options.

**TABLE 8:** Available recommendations for difficult‐to‐treat RRMM subgroups.

Reference	Year published	Available subgroup recommendations
ASCO and CCO [116]	2020	High‐risk CA, renal impairment, extramedullary disease
Mateos et al. [117]	2021	High‐risk CA, frailty
EHA‐ESMO [2]	2021	Renal impairment, extramedullary disease (solitary plasmacytoma)
Zamagni et al. [118]	2022	High‐risk CA, elderly
Li et al. [119]	2022	Extramedullary disease
Pawlyn et al. [120]	2022	Frailty
Bladé et al. [11]	2022	Extramedullary disease
Facon et al. [121]	2023	Elderly, frailty
NCCN [29]	2023	Extramedullary disease (solitary plasmacytoma)

## AUTHOR CONTRIBUTIONS

All authors participated in the drafting, review and editing of the manuscript and in the review and approval of the final version of the manuscript.

## CONFLICT OF INTEREST STATEMENT

**M‐SR**: honoraria – BMS, Janssen; advisory role – Amgen, BMS, Janssen, Novartis, Sanofi, Takeda; consultant – Amgen, BMS, Janssen, Novartis, Sanofi, Takeda; research support – BMS, Janssen, Novartis, Sanofi. **EZ**: honoraria – Amgen, BMS, GSK, Janssen, Pfizer, Roche, Sanofi, Takeda; advisory role – Amgen, BMS, GSK, Janssen, Pfizer, Roche, Sanofi, Takeda. **SM**: advisory role – AbbVie, Adaptive Biotechnology, Amgen, Celgene/BMS, GlaxoSmithKline, Janssen, Novartis, Oncopeptide, Regeneron, Roche, Sanofi, Takeda; research support – AbbVie, Adaptive Biotechnology, Amgen, Celgene/BMS, GlaxoSmithKline, Janssen, Novartis, Oncopeptide, Regeneron, Roche, Sanofi, Takeda. **PR‐O**:  honoraria – Amgen, BMS, GSK, Janssen, Regeneron, Sanofi, Takeda; advisory role – Abbvie, BMS, GSK, Janssen, Kite Pharma, Oncopeptides, Pfizer, Sanofi, Takeda; consultant – Celgene‐BMS, GSK, Pfizer. **FS**: honoraria – Abbvie, Amgen, BMS, Daiichi‐Sankyo, GSK, Janssen, Novartis, Oncopeptides, Pfizer, Sanofi, SkyliteDX, Takeda; advisory role – Abbvie, Celgene, GSK, Janssen, Oncopeptides, Sanofi, Takeda; research support – Celgene, GSK, Janssen, Oncopeptides, Sanofi, Targovax. **AB**: honoraria – Amgen, BMS, Janssen, Sanofi, Takeda; advisory role – Amgen, BMS, Janssen, Sanofi, Takeda.

## FUNDING INFORMATION

Sanofi

## Data Availability

Data sharing is not applicable to this article as no datasets were generated or analysed during the current study.
